# Co-occurrence of 3 different resistance plasmids in a multi-drug resistant *Cronobacter sakazakii* isolate causing neonatal infections

**DOI:** 10.1080/21505594.2017.1356537

**Published:** 2017-08-16

**Authors:** Lining Shi, Quanhui Liang, Zhe Zhan, Jiao Feng, Yachao Zhao, Yong Chen, Mei Huang, Yigang Tong, Weili Wu, Weijun Chen, Xiaojun Li, Zhe Yin, Jinglin Wang, Dongsheng Zhou

**Affiliations:** a Institute of Medical Laboratory Sciences, Jinling Hospital, School of Medicine, Nanjing University, Nanjing, China; b Department of Clinical Laboratory, The First People's Hospital of Foshan, Foshan, China; c State Key Laboratory of Pathogen and Biosecurity, Beijing Institute of Microbiology and Epidemiology, Beijing, China; d Beijing Institute of Genomics, Chinese Academy of Sciences, Beijing, China

**Keywords:** carbapenem resistance, *Cronobacter sakazakii*, multi-drug resistance, plasmids

## Abstract

*Cronobacter sakazakii* 505108 was isolated from a sputum specimen of a neonate with severe pneumonia. *C. sakazakii* 505108 co-harbors 3 resistance plasmids of the IncHI2, IncX3, and IncFIB incomparability groups, respectively. These 3 plasmids have acquired several accessory modules, which carry an extremely large number of resistance genes, especially including those involved in resistance to carbapenems, aminoglycoside, tetracyclines, and phenicols and sulphonamide/trimethoprim. These plasmid-borne antibiotic resistance genes were associated with insertion sequences, integrons, and transposons, indicating that the assembly and mobilization of the corresponding accessory modules with complex chimera structures are facilitated by transposition and/or homologous recombination. This is the first report of fully sequence plasmids in clinical *Cronobacter*, which provides a deeper insight into plasmid-mediated multi-drug resistance in *Cronobacter* from hospital settings.

## Introduction

*Enterobacter sakazakii* was initially defined in 1980 and reclassified into a new genus *Cronobacter* in 2007,^1^ currently composed of 7 species.^2^ *Cronobacter* species are motile, non-sporeforming, peritrichous rods within the *Enterobacteriaceae* family and ubiquitously distributed in nature. *Cronobacter* can cause serious infections in neonates and infants, especially those premature or with low birth weight,^3-5^ and infections in elderly and immunocompromised adults have also been reported.^6,7^ *C. sakazakii, C. malonaticus* and *C. turicensis* are considered as opportunistic human pathogens and account for the majority of clinical isolates of *Cronobacter.*^3-5^ *Cronobacter*-induced neonatal infections manifest as necrotizing enterocolitis, meningitis, septicaemia and severe pneumonia with mortality rates of 40–80%, and in most cases are epidemiologically associated with ingestion of contaminated powdered infant formula.^3-5^ The number of *Cronobacter* infection cases is underestimated due to misidentification of *Cronobacter* as other species such as *Enterobacter cloacae*.

*Cronobacter* isolates are generally susceptible to the most commonly clinically used antimicrobial agents, but resistance to one or more old-generation antimicrobials such as cephalothin, streptomycin, gentamicin and tetracycline has developed in a few *Cronobacter* isolates.^8,9^ The production of chromosomal AmpC β-lactamases, including CSA-1 and CSA-2 in *C. sakazakii*, and CMA-1 and CMA-2 in *C. malonaticus*, confers the resistance exclusively to the first generation cephalosporins (e.g. cephalothin).^10^ The *tetA(B)* gene and additional unknown determinants for tetracycline resistance have been reported in an environmental tetracycline-resistant *Cronobacter* isolate.^11^ A multi-drug resistant (MDR) *C. sakazakii* isolate of animal origin co-harbors an IncI2 plasmid pWF-5–19C_mcr-1 (accession number KX505142) carrying *mcr-1* (colistin resistance) and an IncB/O plasmid pWF-5–19C_NDM (accession number KX505142) containing *fosA3* (fosfomycin resistance) and *bla*^NDM-9^ (carbapenem resistance).^12^ pWF-5–19C_NDM is partially sequenced, while pWF-5–19C_mcr-1 represents the single fully sequenced antibiotic resistance plasmid in *Cronobacter*.

Co-existence of *bla*_VEB-1_ (extended-cephalosporin resistance), *qnrA* (quinolone resistance) and *arr-2* (rifampin resistance) in a plasmid-borne class 1 integron has been identified in a nosocomial MDR *Cronobacter* isolate.^13,14^ These examples represent the few reports of plasmid-mediated MDR in clinical *Cronobacter* isolates, but neither the integron nor the plasmid has been fully sequenced.

This study deals with detailed genetic characterization of 3 resistance plasmids co-existing in a MDR *C. sakazakii* isolate causing severe neonatal pneumonia. These 3 plasmids carry a total of 22 non-redundant genes or gene loci involved in resistance to antimicrobials and heavy metals. This is the first report of fully sequenced antibiotic resistance plasmids in *Cronobacter* of clinical origin.

## Results and discussion

### Case report

On April 28 2016, a female neonate with hyperpyrexia, bradypsychia, hyperspasmia, refusal to feed, recurrent apnea and severe skin jaundice was hospitalized in a public children's hospital in Nanjing City, China, and diagnosed to have bilirubin encephalopathy accompanied with severe pneumonia. Once hospitalized, the patient received a series of symptomatic treatments, especially including nutrition support therapy, exchange transfusion, neonatal phototherapy, mechanical ventilation; in addition, empirical intravenous antimicrobial treatment with latamoxef. Bacterial isolates were repeatedly recovered from the sputum specimens during routine sampling and cultivation from April 30th to May 5th, and one of these isolates was designated 505108. Based on the antimicrobial susceptibility test results, the antibiotic therapy was switched to intravenous administration with erythromycin since May 1st. Her symptoms associated with bilirubin encephalopathy and pneumonia gradually improved.

### *C. sakazakii* co-harboring 3 resistance plasmids

PCR detection, followed by PCR amplicon sequencing, disclosed that the 505108 isolate carried the 2 *C. sakazakii* signature sequences of *cgcA* and *gyrB*. The multilocus sequence typing (MSLT) showed that the 505108 isolate belonged to the *C. sakazakii* sequence type 1 (ST1), with an allelic profile 1–1–1–1–1–1–1 corresponding to the 7 housekeeping gens *atpD, fusA, glnS, gltB, gyrB, infB* and *pps*. PCR screening indicated the presence of *bla*_NDM-1_, but none of the other carbapenemase genes were found in *C. sakazakii* 505108.

The 505108 isolate was found to harbor 3 plasmids, designated p505108-MDR, p505108-NDM and p505108-T6SS, which had circularly closed DNA sequences of 312,880 bp, 53,793 bp and 139,553 bp in length with mean G+C contents of 47.7%, 49.0% and 56.4%, and contained 359, 62 and 126 predicted open reading frames (ORFs), respectively (Figure S1 and Table 1).

**Table 1.** Major features of plasmids analyzed.

	Plasmids					
Category	p505108-MDR	R478^@^	p505108-NDM	pNDM-HN380^@^	p505108-T6SS	pESA3^@^
Incomparability group	IncHI2	IncHI2	IncX3	IncX3	IncFIB	IncFIB
Total length (bp)	312,880	274,762	53,793	54,035	139,553	131,196
Total number of ORFs	359	304	61	62	126	117
Mean G+C content, %	47.7	45.5	49.0	49.0	56.4	56.8
Length of the backbone (bp)	207,144	212,499	34,700	34,732	131,195	131,196
Accessory modules	The MDR-1 region^#^, the MDR-2 region^#^, Tn*6362*^#^, the *aphA1a* region^#^, Tn*2*^#^, the IS*Cfr9*-IS*Cfr15* region, and 2 separate copies of ΔIS*903D*	The Tn*1696*-Tn*6322* region^#^, The *sil-cop* region^#^, Tn*10*^#^, IS*186B*, and IS*150*	The *bla*_NDM-1_ region^#^, and IS*Kox3*		The *aphA1a* region^#^	None

Note.

@ reference plasmids included in genomic comparison;

# accessory modules containing resistance genes as listed in Table 2.

Each plasmid was composed of the backbone regions, together with the accessory modules that were recognized as acquired DNA regions associated with and bordered by mobile elements and inserted at different sites of the backbone (Figure S1). A total of 22 non-redundant genes or gene loci, which were involved in resistance to antimicrobials (β-lactams including carbapenems, quinolons, aminoglycosides, tetracyclines, phenicols, sulphonamides, trimethoprims, rifampicins, bleomycin and acriflavin) and heavy metals (arsenic, copper, mercury, nickel/cobalt and tellurium), were found not only in the accessory modules but also in the backbones of these 3 plasmids (Table 1 and 2).

**Table 2.** Drug resistance genes in sequenced plasmids.

Plasmid	Resistance marker	Resistance phenotype	Nucleotide position	Region located
p505108-MDR	The *ter* locus	Tellurium resistance	62591..82538	The plasmid backbone
	The *ars* locus	Arsenic resistance	156203..159087	
	*dfrA18*	Trimethoprim resistance	124249..124818	The MDR-1 region
	*strAB*	Aminoglycoside resistance	126592.. 128231	
	The *rcn* locus	Nickel/cobalt resistance	142132..143642	
	*bla*_SHV-12_	β-lactam resistance	239239..240099	The MDR-2 region
	*bla*_DHA-1_	β-lactam resistance	247503..248642	
	*aacC3*	Aminoglycoside resistance	265953..266762	
	*aacA27*	Aminoglycoside resistance	268847..269428	
	*aacA4cr*	Quinolone and aminoglycoside resistance	235221..235775	
	*qnrB4*	Quinolone resistance	252763..253410	
	*tetA*(D)	Tetracycline resistance	224408..225592	
	*catA2*	Phenicol resistance	220639..221280	
	*sul1*	Sulphonamide resistance	245087..245926	
	*sul1*	Sulphonamide resistance	262128..262967	
	*arr7*	Rifampicin resistance	265412..265807	
	The *mer* locus	Mercuric resistance	102624..106878	Tn*6362*
	*aphA1a*	Aminoglycoside resistance	164776..165591	The *aphA1a* region
	*bla*_TEM-1B_	β-lactam resistance	113444..114304	Tn*2*
p505108-NDM	*bla*_NDM-1_	Carbapenem resistance	17827..18639	The *bla*_NDM-1_ region
	*bla*_SHV-12_	β-lactam resistance	9324..10184	
	*ble*_MBL_	Bleomycin resistance	17560..17925	
p505108-T6SS	*acrAB*	Acriflavin resistance	13799..17998	The plasmid backbone
	The *ars* locus	Arsenic resistance	47576..49740	
	*scsAB*	Copper resistance	70758..73326	
	*aphA1a*	Aminoglycoside resistance	77928..78743	The *aphA1a* region

p505108-NDM could be transferred into *E. coli* through conjugation, generating the transconjugant 505108-NDM-EC600 (Table 3). Repeated attempts failed to transfer p505108-MDR or p505108-T6SS into *E. coli* through conjugation and electroporation. Class B carbapenemase activity was observed in both 505108 and 505108-NDM-EC600 (data not shown), which was due to production of NDM enzyme in these 2 strains.

**Table 3.** Antimicrobial drug susceptibility profiles.

		MIC (mg/L) /antimicrobial susceptibility		
Category	Antibiotics	505108	505108-NDM-EC600	EC600
Penicillin	Ampicillin	>1024/R	>1024/R	<4/S
Cephalosporin	Ceftazidime	>512/R	>512/R	<4/S
Carbapenem	Meropenem	16/R	8/R	<1/S
Cephamycin	Cefoxitin	512/R	128/R	<8/S
Monobactam	Aztreonam	128/R	128/R	<4/S
Aminoglycoside	Amikacin	1024/R	<8/S	<8/S
Tetracycline	Minocycline	32/R	<1/S	<1/S
Phenicol	Chloramphenicol	512/R	<8/S	<8/S
Folate pathway Inhibitors	Trimethoprim	>32/R	<0.25/S	<0.25/S
	Sulfamethoxazole	608/R	4.75/S	4.75/S
Nitrofuran	Nitrofurantoin	32/S	16/S	8/S
Fluoroquinolone	Ciprofloxacin	<1/S	<1/S	<1/S
Macrolide	Azithromycin	8/S	<4/S	4/S
Fosfomycin	Fosfomycin	<64/S	<64/S	<64/S
Glycylcycline	Tigecycline	<1/S	<1/S	<1/S
Lipopeptide	Colistin	<1/S	<1/S	<1/S

*Note.* S=sensitive; R=resistant.

The 505108 isolate was resistant to ampicillin, ceftazidime, meropenem, cefoxitin, aztreonam, amikacin, minocycline, chloramphenicol, trimethoprim and sulfamethoxazole, but remained susceptible to nitrofurantoin, ciprofloxacin, azithromycin, fosfomycin, tigecycline and colistin; as expected, the transconjugant 505108-NDM-EC600 was resistant to ampicillin, ceftazidime, meropenem, cefoxitin and aztreonam, but remained susceptible to all the other drugs tested (Table 3).

### General features of p505108-MDR

The p505108-MDR backbone had 95% BLAST query coverage and 99% nucleotide identity to the reference IncHI2 plasmid R478,^15^ and these 2 plasmids shared the core IncHI2 backbone markers including *repHI2A* and *repHI2B* for replication initiation, *parAB* and *parMR* for partition, and the *tra1* and *tra2* regions for conjugal transfer (Figure S1 and S2).

Whole genome comparison of p505108-MDR and R478 disclosed 10 different regions (DFRs), designated DFR-1 to DFR-10 (Figure S2). A ΔIS*903D* element (DFR-1) was inserted between *parR* and *htdA* within the *tra2* region of p505108-MDR, probably making p505108-MDR non-conjugative. DFR-2 was located between *orf564* and *orf312* and organized as the *hipB* to *orf411* backbone region, Tn*6362*, the *orf189* to *orf258* backbone region in p505108-MDR, but manifested as the Tn*1696*-Tn*6322* region in R478; the acquisition of Tn*1696*-Tn*6322* resulted in the loss of the above 2 small backbone regions from R478. Tn*2* (DRF-3) was inserted into *orf159* (splitting it into 2 separate parts) in p505108-MDR, which left 5-bp direct repeats (DRs; target site duplication signals of transposition) at both ends of Tn*2*. DFR-4 existed as the 11.4-kb *sil-cop* region (conferring resistance to silver and copper) that was inserted between the 2 backbone genes *orf159* and *orf819* in R478, but as the 25-kb MDR-1 region in p505108-MDR. The 6.9-kb *aphA1a* region (DRF-5) was observed between *int* and *mucAB* in p505108-MDR, and its acquisition led to truncation of *mucA* and loss of the *orf318* to *retA* region.

In R478, the class C tetracycline resistance transposon Tn*10* (DRF-6) was inserted into *orf300*, while the IS*186B* element (DRF-7) existed between *orf321* and *ldrB*. DRF-8 was composed of the *relE* to *orf612* region and the IS*Cfr9*-IS*Cfr15* region (both of which lacked resistance genes) in p505108-MDR, while it existed as an IS*150* element flanked by 5-bp DRs in R478. The acquisition of IS*Cfr9*-IS*Cfr15* by p505108-MDR and that of IS*150* by R478 led to truncation of downstream *orf606* and loss of the upstream *relE* to *orf612* region, respectively. The 52.4 kb MDR-2 region (DFR-9) was inserted into *klaB* in p505108-MDR, leading to truncation of *klaB* as well as deletion of the downstream *klaA-orf609* region. A second copy of IS*903D* (DFR-10) was inserted between *orf2385* and *orf450*, leaving both of them truncated.

DFR-1, DFR-3 to DFR-7 and DFR-10 were entirely composed of accessory modules, while the other DFRs consisted of not only accessory modules but backbone regions; the acquisition of accessory modules induced deletion and/or truncation of surrounding backbone regions (Figure S2). Although p505108-MDR and R478 shared the overwhelming majority of their backbones, these 2 plasmids carried different profiles of accessory modules, most of which were inserted at different sites across the plasmid backbones.

**Figure 1.**
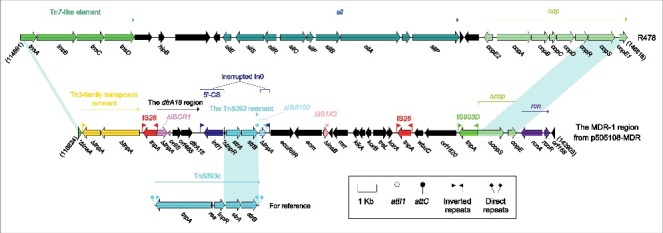
The MDR-1 region from p505108-MDR and comparison with related regions. Genes are denoted by arrows. Genes, mobile elements and other features are colored based on function classification. Shading denotes regions of homology (> 95% nucleotide identity). Numbers in brackets indicate the nucleotide positions within the corresponding plasmids.

**Figure 2.**
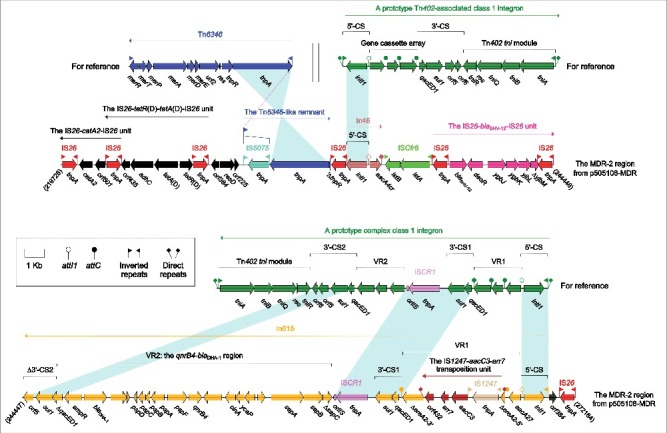
The MDR-2 region from p505108-MDR and comparison with related regions. Genes are denoted by arrows. Genes, mobile elements and other features are colored based on function classification. Shading denotes regions of homology (> 95% nucleotide identity). Numbers in brackets indicate the nucleotide positions within the corresponding plasmids.

**Figure 3.**
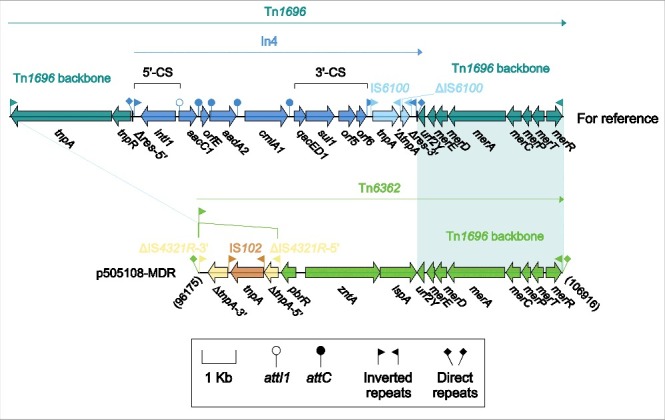
Tn*6362* from p505108-MDR and comparison with related regions. Genes are denoted by arrows. Genes, mobile elements and other features are colored based on function classification. Shading denotes regions of homology (> 95% nucleotide identity). Numbers in brackets indicate the nucleotide positions within the corresponding plasmids.

**Figure 4.**
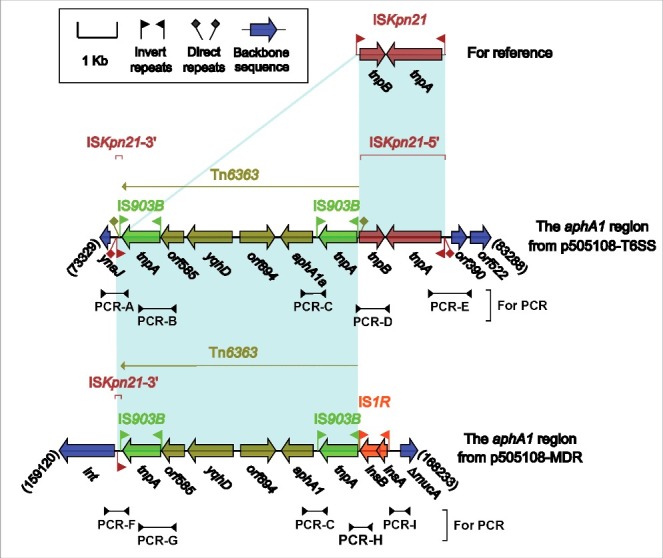
The *aphA1a* regions from p505108-MDR and p505108-T6SS and comparison with related regions. Genes are denoted by arrows. Genes, mobile elements and other features are colored based on function classification. Shading denotes regions of homology (> 95% nucleotide identity). Numbers in brackets indicate the nucleotide positions within the corresponding plasmids. The arrowheads indicated the location of PCR primers and the expected amplicons. See Figure S6 for the PCR results.

There were in total 5 accessory modules containing resistance genes in p505108-MDR, namely the MDR-1 region (Fig. 1), the MDR-2 region (Fig. 2), Tn*6362* (Fig. 3), the *aphA1a* region (Fig. 4) and the *bla*_TEM-1B_-carrying Tn*2* (Figure S4).

### The MDR-1 region from p505108-MDR

The MDR-1 region (Fig. 1) was a derivative of, although dramatically genetically differed from, the *sil-cop* region of R478 because these 2 modules were located at the same site of the IncHI2 backbone and had the same terminal regions. The *sil-cop* region of R478 carried a Tn*7*-like core transposition module *tnsABCD* and the silver (*sil*) and copper (*cop*) resistance loci. Various derivatives of the *sil-cop* region were found in several other IncHI2 plasmids.^16^

Being dramatically distinct from the *sil-cop* region of R478, the MDR-1 region lost the entire *sil* and the most parts of *tnsABCD* and *cop*, but instead acquired several intact or residue mobile elements associated with resistance genes, especially including the *dfrA18* region and an unusual In0 with paired terminal inverted repeats (TIRs). The *dfrA* genes were often associated with IS*CR1*^17^ as observed in the *dfrA18* region of p505108-MDR, in which IS*CR1* was truncated due to its connection with upstream IS*26*. The prototype In0 was an empty class 1 integron, but a 1.9-kb Tn*5393* remnant carrying the streptomycin resistance module *strAB* was integrated at a site downstream of the PcW_TGN-10_ promoter of In0 in p505108-MDR, interrupting In0 into 2 separate parts.

### The MDR-2 region from p505108-MDR

The MDR-2 region (Fig. 2) had a complex chimera structure, which carried 5 resistance-conferring substructures, namely the chloramphenicol resistance unit IS*26-catA2*-IS*26*, the tetracycline resistance unit IS*26-tetR*(D)*-tetA*(D)-IS*26* [also designated Tn*tetD*^18^], the extended-spectrum β-lactam resistance unit IS*26-bla*_SHV-12_*-*IS*26*, In46 and In615. The 3 IS*26*-flanking units lacked short DR sequences at both ends and were identified as IS*26*-composite transposon-like mobile elements.^19-21^ A total of 7 copies of IS*26* were found in the MDR-2 region, and the common IS*26* component acts as an adaptor to mediate massive recombination and transposition events and thereby plays a pivotal role in assembly of large MDR regions with complex mosaic structures.^22,23^

In46 from the MDR-2 region contained the 5′-conserved segment (5′-CS) and the sole gene cassette *aacA4cr*, but lacked the inverted repeat at the integrase end (IRi) and the 3′-terminal region composed of 3′-CS and inverted repeat at the *tni* end (IRt), which was resulted from connection of In46 with upstream IS*26* and downstream IS*Cfr8*. In615 was a complex class 1 integron that contained 2 resistance gene-carrying variable regions (VRs: VR1 and VR2). IS*CR1* mobilized the nearby VR1 together with 3′-CS2 from one integron to 3′-CS1 of another integron, facilitating the formation of complex class 1 integrons.^17^ The primary structure of VR1 from In615 was the gene cassette array *aacA27-ereA2*, in which *ereA2* was interrupted into 2 separate parts by insertion of the IS*1247-aacC3-arr7* transposition unit with 4-bp DRs at both ends.^24^ The *qnrB4-bla*_DHA-1_ region (VR2) connected with IS*CR1* was found several complex class 1 integrons carried on plasmids, including pCFI-1 (accession number JN215523), pCFI-2 (accession number JN215524), pCFI-3 (accession number JQ356870), pNMDHA (accession number GU943791), pRBDHA (accession number AJ971343) and pMPDHA (accession number AJ971344).^25^

### Tn6362 from p505108-MDR

Tn*1696* (Fig. 3), a unit transposon of the Tn*21* subgroup of Tn3 family, was assembled from insertion of class 1 integron In4 into the resolution (*res*) site of a backbone structure IRL (inverted repeat left)-*tnpA* (transposase)-*tnpR* (resolvase)-*res-mer*-IRR (inverted repeat right).^26^ As a derivative of Tn*1696*, Tn*6362* (Fig. 3) retained the *mer*-IRR region but had 2 major modifications: i) IRL was interrupted by IS*4321R* (the IS*1111* family members IS*4321* and IS*5075* target and are inserted at a specific position in the 38-bp TIRs of Tn*21* subgroup transposons),^27^ which was further interrupted by IS*102*; and ii) a *pbrR-zntA-lspA* region, probably involved in zinc uptake, was acquired instead of the *tnpAR-res*:In4 region. Tn*6362* was bracketed by 5-bp DRs, indicating that mobilization of Tn*6362* into p505108-MDR was a transposition process requiring the core transposition determinants (TIRs, *tnpAR* and *res*), and that the lesion or loss of these core determinants occurred post transposition.

### Comparison of p505108-NDM with closely related pNDM-HN380

pP10159–1 showed >99% BLAST query coverage and >99% nucleotide identity to the first fully sequenced *bla*_NDM_-carrying IncX3 plasmid pNDM-HN380.^28^ These 2 plasmids harbored 2 accessory modules, namely an IS*Kox3* element and an approximately 18-kb *bla*_NDM-1_ region containing 3 resistance genes *bla*_NDM-1_, *ble*_MBL_ and *bla*_SHV-12_ (Figure S4). The *bla*_NDM-1_ regions of p505108-NDM and pNDM-HN380 might be generated from connection of the prototype *bla*_NDM-1_-carrying IS*Aba125*-flanked composite transposon Tn*125*^29^ with the upstream IS*3000*-ΔTn*3* region and the downstream composite transposon-like IS*26-bla*_SHV-12_-IS*26* unit,^21^ making the truncation of Tn*125* itself; moreover, an IS*5* element was inserted into IS*Aba125* at the 5′-flank of Tn*125*, interrupting IS*Aba125* into 2 separate parts. These 2 *bla*_NDM-1_ regions slightly differed from each other by a 111-bp insertion at adjacent position between IS*26-bla*_SHV-12_-IS*26* and ΔTn*125* and also by a 304-bp deletion within the disrupted IS*Aba125*.

### Comparison of p505108-T6SS with closely related pESA3

p505108-T6SS and pESA3^30^ had almost identical backbones (100% BLAST query coverage and >99% nucleotide identity) and carried a single IncFIB-type replication gene *repA*, the plasmid partition locus *parAB*, the toxin-antitoxin system locus *hipAB* for post-segregational killing, 4 virulence loci [including *cpa* (plasminogen activator; serum resistance and invasion),^31^ *eit* and *iuc* (iron acquisition), and a type 6 secretion system (T6SS) locus] and 2 putative resistance loci *acrAB* and *ars* (Figure S5). The insertion of an 8.3-kb *aphA1a* region (see below) at a site between *ynaJ* and *orf390* in p505108-T6SS accounted for the major modular difference between p505108-T6SS and pESA3 (Figure S5).

### The aphA1a regions from p505108-MDR and p505108-T6SS

The presence of 2 highly similar *aphA1a* regions (Fig. 4) in the 2 co-existent plasmids p505108-MDR and p505108-T6SS were validated, although highly unusual, by a set of PCR amplifications that targeted several key jointing fragments of these 2 *aphA1a* regions and their surrounding backbone regions, using genomic DNA of the 505108 isolate as template.

The *aphA1a* region of p505108-MDR was generated from 2 different transposition events: i) insertion of an IS*Kpn21* element (IRL-*tnpAB*-IRR) into the p505108-MDR backbone, and ii) that of an IS*903B*-flanked composite transposon Tn*6363* carrying *aphA1a* at a site between *tnpB* and IRR of IS*Kpn21*, interrupting IS*Kpn21* 2 separate parts ΔIS*Kpn21*–5′ (IRL-*tnpA-tnpB*) and ΔIS*Kpn21*–3′ (IRR). These 2 transposition events left 5-bp and 9-bp DRs bracketing IS*Kpn21* and Tn*6363*, respectively.

The prototype *aphA1a* region (as observed in p505108-MDR) was likely connected with an IS*1R* element, which resulted from transposition or homologous recombination, generating the *aphA1a* region of p505108-T6SS with deletion of ΔIS*Kpn21*–5′ relative to p505108-MDR. pESA3 and its close derivatives including p505108-T6SS have been widely identified as virulence plasmids in pathogenic *C. sakazakii* strains.^32^ Notably, acquisition of the *aphA1a* region by p505108-T6SS made it to be a carrier of not only virulence determinants but also antibiotic resistance markers.

### Concluding remarks

*Cronobacter* species have the ability to survive in powdered infant formula, and *C. sakazakii, C. malonaticus* and *C. turicensis* represent dangerous opportunistic pathogens of neonates.^33^ *Cronobacter* species tend to be more sensitive to most antibiotics than other *Enterobacteriaceae* species. There are few reports describing the MDR in *Cronobacter* isolates of both environmental and clinical origins, and molecular mechanisms of antimicrobial resistance in *Cronobacter* are poorly understood. *C. sakazakii* 505108, causing severe neonatal pneumonia, co-harbors 3 resistance plasmids belonging to the IncHI2, IncX3 and IncFIB incomparability groups, respectively. These 3 plasmids carry an extremely large number of resistance genes, and most of these plasmid-borne resistance genes were associated with insertion sequences, integrons and transposons, constituting various large accessory modules with chimera structures. Mobilization of these accessory resistance modules into plasmid backbones are promoted by transposition and homologous recombination. MDR in *Cronobacter* isolates leads to limited choice of antibiotics for treatment, resulting in a greater risk of death. Therefore, surveillance of plasmid-mediated MDR in clinical *Cronobacter* isolates is of paramount importance.

## Materials and methods

### Bacterial strains and identification

Bacterial species identification was performed using 16S rRNA gene sequencing^34^ and PCR-detection of a 492-bp *cgcA* sequence^35^ and a 151-bp *gyrB* sequence^36^ specific for *C. sakazakii*. The MLST scheme for *C. sakazakii* was derived from the PubMLST database (https://pubmlst.org/cronobacter/).The major plasmid-borne carbapenemase genes were screened for by PCR.^37^ All the PCR amplicons were sequenced on ABI 3730 Sequencer (LifeTechnologies, CA, USA) with the same primers as used for PCR.

### Sequencing and annotation

Genomic DNA was isolated from the 505108 isolate using a Qiagen large construct kit and sequenced from a mate-pair library with average insert size of 5,000 bp, using a MiSeq sequencer (Illumina, CA, USA). DNA contigs were assembled using Newbler 2.6.^38^ Gaps between contigs were filled using a combination of PCR and Sanger sequencing using an ABI 3730 Sequencer. Open reading frames and pseudogenes were predicted using RAST 2.0^39^ combined with BLASTP/BLASTN^40^ searches against the UniProtKB/Swiss-Prot^41^ and RefSeq^42^ databases. Annotation of resistance genes, mobile elements and other features was performed using CARD,^43^ ResFinder,^44^ ISfinder^45^ and INTEGRALL.^46^ Multiple and pairwise sequence comparisons were performed using MUSCLE 3.8.31^47^ and BLASTN, respectively. Gene organization diagrams were drawn in Inkscape 0.48.1.

### Plasmid transfer

Plasmids were transferred in attempt from the 505108 isolate into *Escherichia coli* TOP10 and EC600 (highly resistant to rifampicin) through electroporation and conjugal transfer, respectively.^48^ For selection of the electroporant or transconjugant containing the markers *repHI2A*+*strA* (p505108-MDR), *repB*+*bla*_NDM_ (p505108-NDM) and *repA*+*aphA1a* (p505108-T6SS), the antibiotics amikacin (20 μg/ml), imipenem (2 μg/ml) and rifampicin (1000 μg/ml) were used in accordance with specific circumstances.

### Phenotypic assays

Activity of Ambler class A/B/D carbapenemases in bacterial cell extracts was determined by a modified CarbaNP test.^48^ Bacterial antimicrobial susceptibility was tested by the broth dilution method and interpreted as per CLSI guidelines.^49^

### Nucleotide sequence accession numbers

The p505108-MDR, p505108-NDM and p505108-T6SS sequences were submitted to GenBank under accession numbers KY978628, KY978629 and KY978630, respectively.

## Supplementary Material

KVIR_S_1356537.zip
