# Supplementary material for: Co-occurrence of 3 different resistance plasmids in a multi-drug resistant Cronobacter sakazakii isolate causing neonatal infections
Source: Virulence. 2017 Aug 16;9(1):110–20. doi: 10.1080/21505594.2017.1356537 (PMC5955447; doi:10.1080/21505594.2017.1356537)
Supplement: KVIR_S_1356537.zip [file kvir-09-01-1356537-s001.zip › KVIR_S_1356537.docx]

**Figure S1|Schematic maps of p505108-MDR, p505108-NDM and p505108-T6SS.** Genes are denoted by arrows, and the backbone and accessory module regions are highlighted in black and color, respectively. The innermost circle presents GC-skew [(G-C)/(G+C)], with a window size of 500 bp and a step size of 20 bp. The next-to-innermost circle presents GC content.

**Figure S2| Linear comparison of p505108-MDR with R478.** Genes are denoted by arrows. Genes, mobile elements and other features are colored based on function classification. Shading denotes regions of homology (>95% nucleotide identity). Numbers in brackets indicate the nucleotide positions within the corresponding plasmids.

**Figure S3|Tn*2* from p505108-MDR.** Genes are denoted by arrows. Genes, mobile elements and other features are colored based on function classification. Numbers in brackets indicate the nucleotide positions within the corresponding plasmids. Tn*2* from p505108-MDR was organized as IRL-*res*-*tnpAR*-*bla*_TEM-1B_-IRR, which was bracketed by 5-bp DRs.

**Figure S4|Comparison of p505108-NDM with pNDM-HN380.** Genes are denoted by arrows. Genes, mobile elements and other features are colored based on function classification. Shading denotes regions of homology (>95% nucleotide identity). Numbers in brackets indicate the nucleotide positions within the corresponding plasmids.

**Figure S5|Linear comparison of p505108-T6SS with pESA3.** Genes are denoted by arrows. Genes, mobile elements and other features are colored based on function classification. Shading denotes regions of homology (>95% nucleotide identity).

**Figure S6|PCR detection of presence of the *aphA1a* regions.** All the nine PCR amplifications, namely PCR-A to PCR-I, gave positive results using genomic DNA of strain 505108 as template. Included were the primer sequences used. See Figure 4 for the location of PCR primers.
