# Supplementary material for: Co-occurrence of 3 different resistance plasmids in a multi-drug resistant Cronobacter sakazakii isolate causing neonatal infections
Source: Virulence. 2017 Aug 16;9(1):110–20. doi: 10.1080/21505594.2017.1356537 (PMC5955447; doi:10.1080/21505594.2017.1356537)
Supplement: KVIR_S_1356537.zip [file kvir-09-01-1356537-s001.zip › 2017VIRULENCE0073R1-s07.pdf]

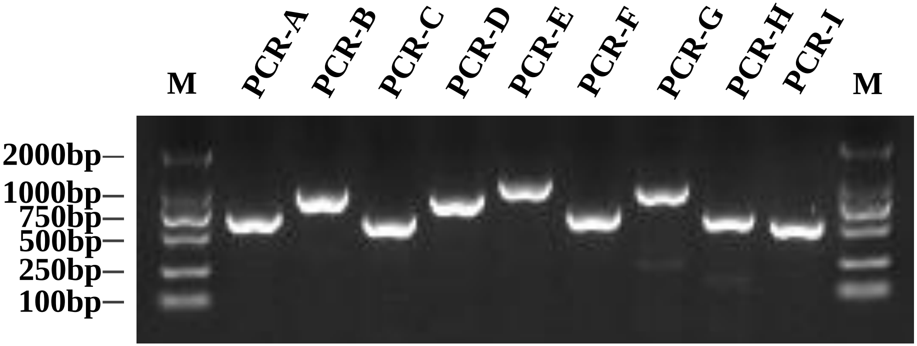

Primers

PCR-A-F1: GATAAACGAGAACGGCACGC
PCR-A-R1: TACAACCGTCGCTCGATAGC
Size of amplicon: 683bp

PCR-B-F1: TCCGTAACGTTGTTCAGCGA
PCR-B-R1: CTATCGTTACCCGGTCGTGG
Size of amplicon: 967bp

PCR-C-F1: GCCGTCAGCCAGTTTAGTCT
PCR-C-R1: AACCGTGCAGTGGCTAATCA
Size of amplicon: 624bp

PCR-D-F1: TCGGGAAGATGCGTGATCTG
PCR-D-R1: CCCAGCGCTCACTGGATAAT
Size of amplicon: 856bp

PCR-E-F1: CCGTGGAGTTCTTCGAGCTT
PCR-E-R1: TCCACAGGAAACCGGCATAC
Size of amplicon: 1087bp

Primers

PCR-F-F1: GCGAATAATGTTCAGCGCGT
PCR-F-R1: TACAACCGTCGCTCGATAGC
Size of amplicon: 645bp

PCR-G-F1: TCCGTCACATTGTTCAGCGA
PCR-G-R1: CTATCGTTACCCGGTCGTGG
Size of amplicon: 967bp

PCR-H-F1: AGTCGTGATGGCAAGGTCAG
PCR-H-R1: TTACGCGTATGACAGGCTCC
Size of amplicon: 599bp

PCR-I-F1: CCTCGCTGAACCTTGCCATA
PCR-I-R1: AACGACGATAGAGCCGTGTC
Size of amplicon: 533bp
