# Supplementary figures and images for: Co-occurrence of 3 different resistance plasmids in a multi-drug resistant Cronobacter sakazakii isolate causing neonatal infections
Source: Virulence. 2017 Aug 16;9(1):110–20. doi: 10.1080/21505594.2017.1356537 (PMC5955447; doi:10.1080/21505594.2017.1356537)

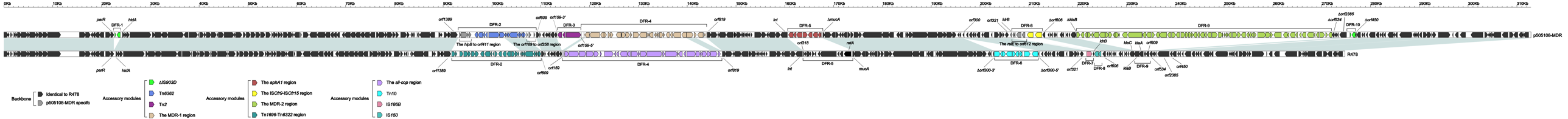

Supplement: KVIR_S_1356537.zip [file kvir-09-01-1356537-s001.zip › 2017VIRULENCE0073R1-s03.pdf]

Tn2

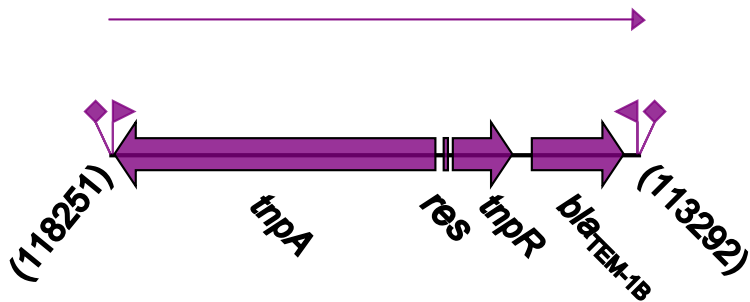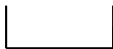

1 Kb

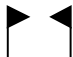

Inverted  
repeats

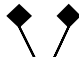

Direct  
repeats

Supplement: KVIR_S_1356537.zip [file kvir-09-01-1356537-s001.zip › 2017VIRULENCE0073R1-s04.pdf]

# a) Linear comparison of sequenced plasmids

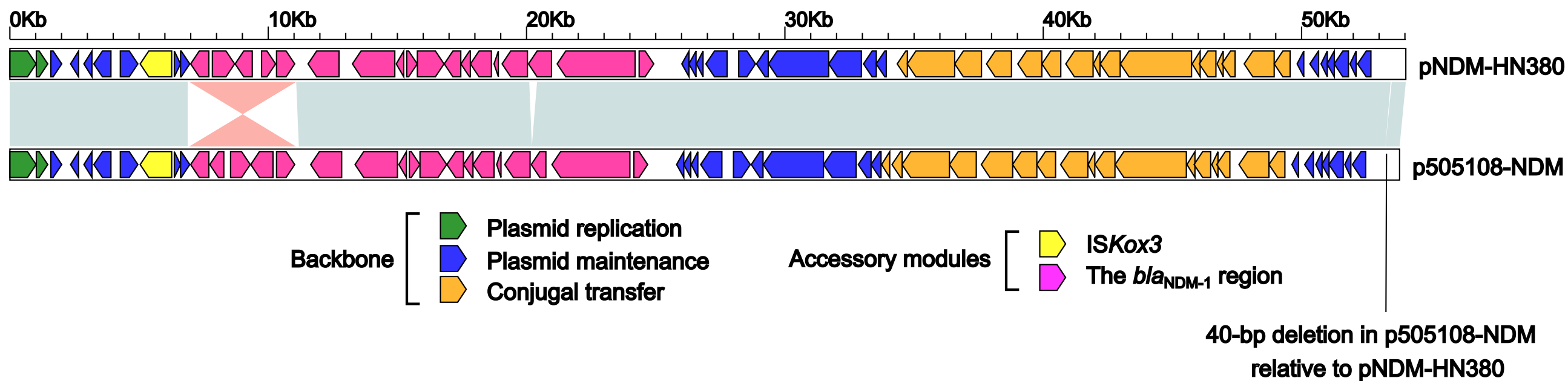

# b) Comparison of the *bla*<sub>NDM-1</sub> regions

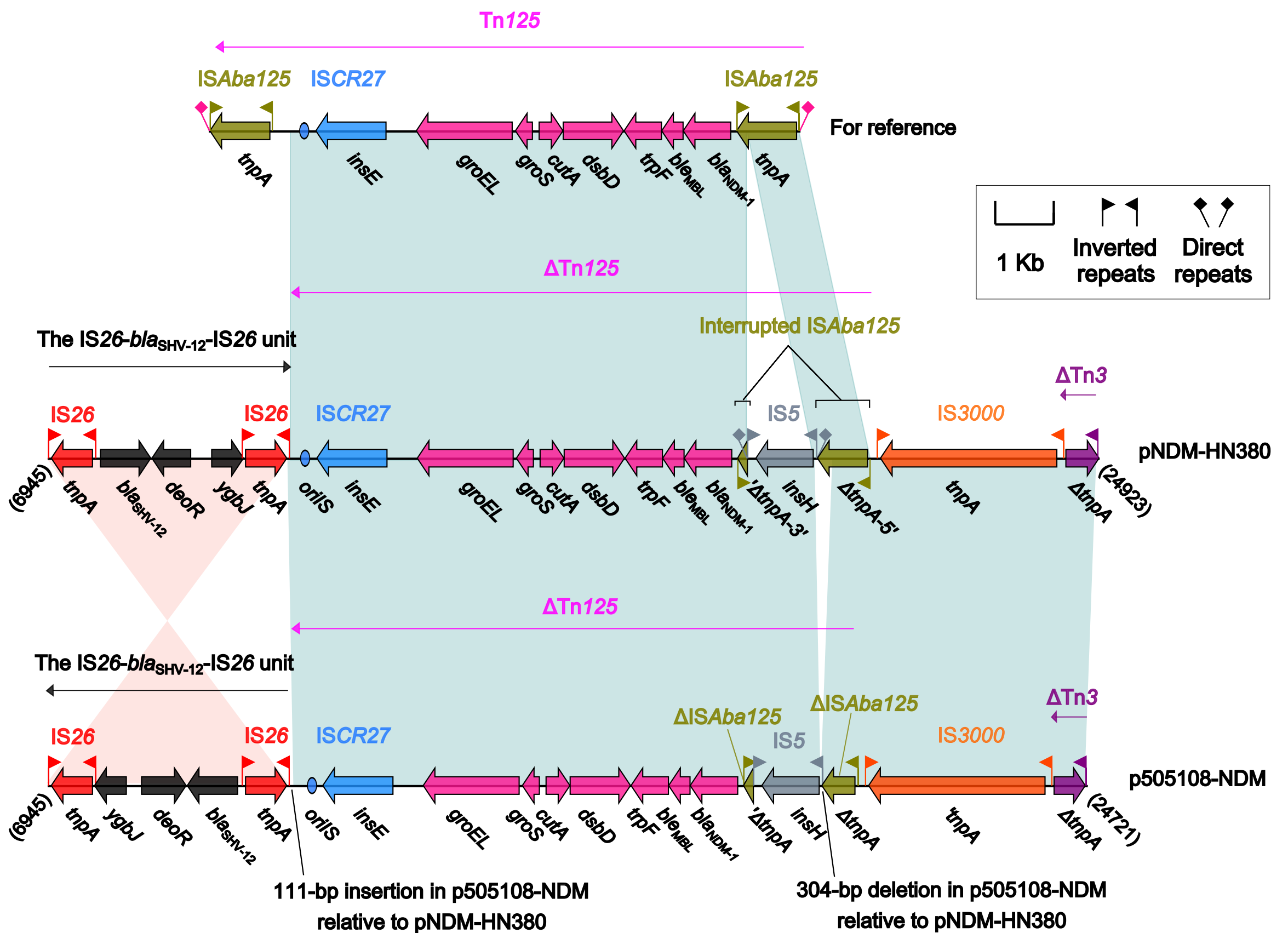

Supplement: KVIR_S_1356537.zip [file kvir-09-01-1356537-s001.zip › 2017VIRULENCE0073R1-s05.pdf]

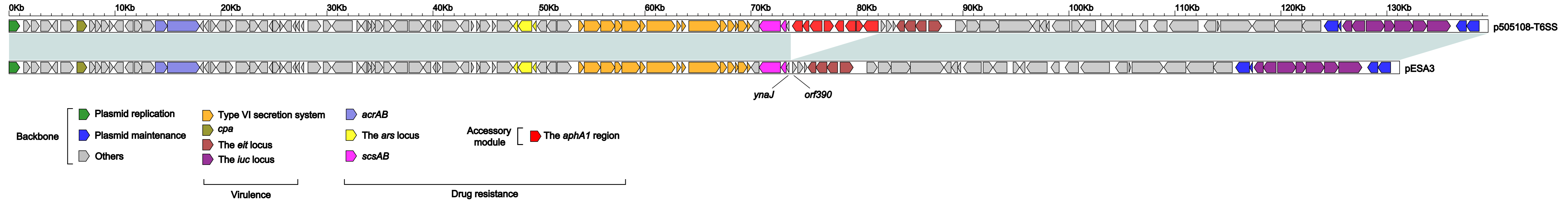

Supplement: KVIR_S_1356537.zip [file kvir-09-01-1356537-s001.zip › 2017VIRULENCE0073R1-s06.pdf]
