# Supplementary material for: Co-occurrence of 3 different resistance plasmids in a multi-drug resistant Cronobacter sakazakii isolate causing neonatal infections
Source: Virulence. 2017 Aug 16;9(1):110–20. doi: 10.1080/21505594.2017.1356537 (PMC5955447; doi:10.1080/21505594.2017.1356537)
Supplement: KVIR_S_1356537.zip [file 2017VIRULENCE0073R1-s02.pdf › 2017VIRULENCE0073R1-s02.pdf]

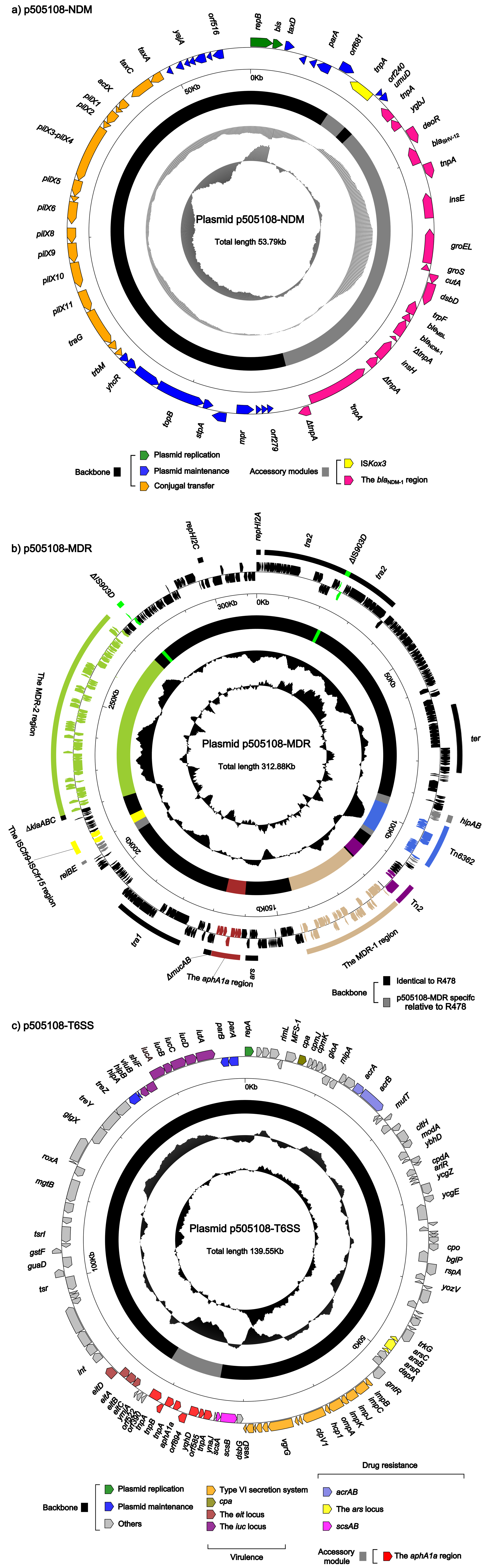

a) p505108-NDM
Plasmid p505108-NDM
Total length 53.79kb
0Kb
50Kb
repB
bis
taxD
parA
orf681
tnpA
orf240
umuD
tnpA
ygbJ
deoR
bla_{SHV-12}
tnpA
insE
groEL
groS
cutA
dsbD
trpF
ble_{MBL}
bla_{NDM-1}
'ΔtnpA
insH
ΔtnpA
tnpA
ΔtnpA
orf279
mpr
stpA
topB
yhcR
trbM
traG
pilX11
pilX10
pilX9
pilX8
pilX6
pilX5
pilX3-pilX4
pilX2
pilX1
actX
taxC
taxA
yajA
orf516
Backbone
Plasmid replication
Plasmid maintenance
Conjugal transfer
Accessory modules
ISKox3
The bla_{NDM-1} region
b) p505108-MDR
Plasmid p505108-MDR
Total length 312.88Kb
0Kb
50Kb
100Kb
150Kb
200Kb
250Kb
300Kb
repHI2C
repHI2A
tra2
ΔIS903D
tra2
ter
hipAB
Tn6362
Tn2
The MDR-1 region
ars
The aphA1a region
ΔmucAB
tra1
relBE
The ISCfr9-ISCfr15 region
ΔklaABC
The MDR-2 region
ΔIS903D
Backbone
Identical to R478
p505108-MDR specifc relative to R478
c) p505108-T6SS
Plasmid p505108-T6SS
Total length 139.55Kb
0Kb
50Kb
100Kb
repA
rimL
MFS-1
cpa
cpmJ
cpmK
gloA
mipA
acrA
acrB
mutT
citH
modA
ybhD
cpdA
ariR
ycgZ
ycgE
cpo
bglP
rspA
yozV
trkG
arsC
arsB
arsR
arsA
gntR
impB
impC
impJ
impK
ompA
hcp1
clpV1
vgrG
vasD
dsbG
scsB
scsA
ynaJ
tnpA
orf585
yqhD
orf894
aphA1a
tnpA
tnpB
tnpA
orf300
orf252
ymjA
eitC
eitB
eitA
eitD
int
tsr
guaD
gstF
tsrl
mgtB
roxA
glgX
treY
treZ
hipA
hipB
vliB
shiF
iucA
iucB
iucC
iucD
iutA
parB
parA
Backbone
Plasmid replication
Plasmid maintenance
Others
Type VI secretion system
cpa
The eit locus
The iuc locus
Virulence
Drug resistance
acrAB
The ars locus
scsAB
Accessory module
The aphA1a region
